# Synthetic biology approaches to actinomycete strain improvement

**DOI:** 10.1093/femsle/fnab060

**Published:** 2021-05-31

**Authors:** Rainer Breitling, Martina Avbelj, Oksana Bilyk, Francesco Del Carratore, Alessandro Filisetti, Erik K R Hanko, Marianna Iorio, Rosario Pérez Redondo, Fernando Reyes, Michelle Rudden, Emmanuele Severi, Lucija Slemc, Kamila Schmidt, Dominic R Whittall, Stefano Donadio, Antonio Rodríguez García, Olga Genilloud, Gregor Kosec, Davide De Lucrezia, Hrvoje Petković, Gavin Thomas, Eriko Takano

**Affiliations:** Department of Chemistry, Manchester Institute of Biotechnology, Manchester Synthetic Biology Research Centre SYNBIOCHEM, The University of Manchester, 131 Princess Street, Manchester, M1 7DN, UK; Biotechnical Faculty, University of Ljubljana, Jamnikarjeva 101, 1000 Ljubljana, Slovenia; Department of Chemistry, Manchester Institute of Biotechnology, Manchester Synthetic Biology Research Centre SYNBIOCHEM, The University of Manchester, 131 Princess Street, Manchester, M1 7DN, UK; Department of Chemistry, Manchester Institute of Biotechnology, Manchester Synthetic Biology Research Centre SYNBIOCHEM, The University of Manchester, 131 Princess Street, Manchester, M1 7DN, UK; Explora Biotech Srl, Doulix business unit, Via Torino 107, 30133 Venice, Italy; Department of Chemistry, Manchester Institute of Biotechnology, Manchester Synthetic Biology Research Centre SYNBIOCHEM, The University of Manchester, 131 Princess Street, Manchester, M1 7DN, UK; Naicons srl., Viale Ortles 22/4, 20139 Milano, Italy; INBIOTEC Instituto de Biotecnología de León, Avenida Real 1, 24006 León, Spain; Fundación MEDINA, Centro de Excelencia en Investigación de Medicamentos Innovadores en Andalucía, Avenida del Conocimiento 34, Parque Tecnologico de Ciencias de la Salud, 18016 Armilla, Granada, Spain; Department of Biology, University of York, Wentworth Way, York, YO10 5DD, UK; Department of Biology, University of York, Wentworth Way, York, YO10 5DD, UK; Biotechnical Faculty, University of Ljubljana, Jamnikarjeva 101, 1000 Ljubljana, Slovenia; Department of Chemistry, Manchester Institute of Biotechnology, Manchester Synthetic Biology Research Centre SYNBIOCHEM, The University of Manchester, 131 Princess Street, Manchester, M1 7DN, UK; Department of Chemistry, Manchester Institute of Biotechnology, Manchester Synthetic Biology Research Centre SYNBIOCHEM, The University of Manchester, 131 Princess Street, Manchester, M1 7DN, UK; Naicons srl., Viale Ortles 22/4, 20139 Milano, Italy; INBIOTEC Instituto de Biotecnología de León, Avenida Real 1, 24006 León, Spain; Fundación MEDINA, Centro de Excelencia en Investigación de Medicamentos Innovadores en Andalucía, Avenida del Conocimiento 34, Parque Tecnologico de Ciencias de la Salud, 18016 Armilla, Granada, Spain; Acies Bio d.o.o., Tehnološki Park 21, 1000, Ljubljana, Slovenia; Explora Biotech Srl, Doulix business unit, Via Torino 107, 30133 Venice, Italy; Biotechnical Faculty, University of Ljubljana, Jamnikarjeva 101, 1000 Ljubljana, Slovenia; Department of Biology, University of York, Wentworth Way, York, YO10 5DD, UK; Department of Chemistry, Manchester Institute of Biotechnology, Manchester Synthetic Biology Research Centre SYNBIOCHEM, The University of Manchester, 131 Princess Street, Manchester, M1 7DN, UK

**Keywords:** actinomycete, synthetic biology, actinobacteria, biosynthesis, biotechnology

## Abstract

Their biochemical versatility and biotechnological importance make actinomycete bacteria attractive targets for ambitious genetic engineering using the toolkit of synthetic biology. But their complex biology also poses unique challenges. This mini review discusses some of the recent advances in synthetic biology approaches from an actinomycete perspective and presents examples of their application to the rational improvement of industrially relevant strains.

## Teaser

Recent advances in synthetic biology driven engineering of the actinomycetes for the production of valuable drug compounds.

## INTRODUCTION

Actinomycetes are chemically extremely talented bacteria, known for the exceptional diversity of their secondary metabolites (Barka *et al*. 2016; Jakubiec-Krzesniak *et al*. 2018). They are a major source for bioactive natural products, serving as starting points for clinically important drugs, in particular antibiotics (Genilloud 2017). Genome analyses show that individual strains of actinobacterial genomes typically contain the biosynthetic machinery for several dozen secondary metabolites, encoded in biosynthetic gene clusters in the genome ready for horizontal gene transfer as a compact functional unit (Lee *et al*. 2020). The emergence of advanced genome engineering techniques and the concepts and methodology of synthetic biology have stimulated renewed interest in exploiting the biosynthetic capabilities of actinomycetes, modifying and improving their performance as biotechnological sources of valuable drugs and drug precursors (Lee *et al*. 2019; Palazzotto *et al*. 2019; Zhao, Wang and Luo 2019). In this mini review, we highlight selected areas of synthetic biology as applied to actinomycete strain improvement. We focus on some areas of recent advances, as well as specific challenges encountered when engineering this specific group of microbes and offer an industrial perspective on the potential applications of synthetic biology to enhance the potential of actinobacteria as drug production hosts.

## THE CHALLENGES OF HETEROLOGOUS EXPRESSION IN ACTINOMYCETES

Novel actinomycete strains, producing new compounds of interest with the potential of becoming industrial products, such as pharmaceuticals, crop-protecting agents or food additives are identified regularly (Onaka 2017; Kim *et al*. 2021). Initially, these novel strains of interest are not immediately used at an industrial scale, as their use presents several challenges due to a limited understanding of the strain and/or the product. The strain could have been identified after bioprospecting (Genilloud 2019; Nogawa, Lopez and Osada 2019) or after expression of a suitable DNA library (Zhang, Tang and Moore 2019). To translate the initial discovery into an industrial product, we need to study the producing strain and the biosynthetic pathway in considerable detail. The methods of synthetic biology are essential for this process. In particular, it is often necessary to genetically engineer the pathway or the host system by the introduction of heterologous genetic material.

Great progress has been made over the past decades in developing gene transfer systems for many actinomycete species and genera (Musiol-Kroll *et al*. 2019; Mitousis, Thoma and Musiol-Kroll 2020), and in devising effective genome editing tools (Alberti and Corre 2019). However, any actinomycete strain newly identified after bioprospecting is, by definition, poorly characterized and consequently often difficult to manipulate genetically. One can look at taxonomically related strains for inspiration; however, as a rule of thumb, developing a gene transfer system for a new strain is an unpredictable endeavor: successful introduction of heterologous DNA can either occur after a few months’ effort, or a whole year can go by without progress. Even after a gene transfer system has been developed for a model strain, the genetic tools available in the actinomycete community will need to be tested and validated in the new strain.

A conceptually attractive alternative to developing a gene transfer system is to rely on production of the desired product in a robust and easy to manipulate host, after heterologous expression of the necessary genes (Xu and Wright 2019; Kang and Kim 2021). This is a relatively straightforward approach that can benefit from a large selection of tools for introducing small and large segments in the host of choice (Tocchetti, Donadio and Sosio 2018), and successful heterologous expression can be confirmed relatively quickly. However, we still know very little about what controls formation of a heterologous product, from supply of precursors to enzymatic cofactors and from regulatory networks to efficient export of the final product. Consequently, heterologous expression of the native biosynthetic gene cluster (BGC) usually results in product titres lower than those obtained in the original host. These challenges necessitate a concerted approach at genetic engineering to optimize both the host production strain and the biosynthetic pathway itself for function in its new context.

## PREDICTIVE MODELLING: METABOLISM AND BEYOND

Predictive metabolic modelling is a key ingredient in the rational engineering of improved production strains (Simeonidis and Price 2015). Such models can help identify competing metabolic pathways, optimal medium design and strategies for selecting high-producing mutants. Stoichiometric constraint-based models of metabolism have been particularly popular and successful for this purpose (Kim, Kim and Lee 2017; Gu *et al*. 2019), and multiple generations of increasingly refined and carefully curated models have been constructed for several important actinomycetes, including the model organisms *Streptomyces coelicolor* (Amara, Takano and Breitling 2018; Kumelj *et al*. 2019), *S. lividans* (D'Huys *et al*. 2012; Valverde, Gullón and Mellado 2018), and the industrial producer *S. clavuligerus* (Medema *et al*. 2010; Toro *et al*. 2018). In each of these, the models have been used not only to understand the basic biology of metabolism, but also to identify targets for strain engineering (Kim, Rocha and Maia 2018).

The availability of well-curated reference models for different *Streptomyces* species has meant that this group could be employed as a benchmark for automated model reconstruction methods (Wang *et al*. 2018). Automated model reconstruction has rapidly expanded the range of actinomycetes for which genome-scale models are available. A large-scale comparative study of genome-scale metabolic models of 37 species of actinobacteria identified conserved core networks of metabolism (Alam *et al*. 2011). Comparative modelling simultaneously optimizing compound biosynthesis and biomass production has been employed to highlight the differences in metabolic capacity for selected classes of secondary metabolites in different potential actinomycete hosts (Zakrzewski *et al*. 2012). More recently, stoichiometric metabolic models have been complemented with enzyme level constraints to obtain more precise predictions of engineering strategies for enhanced secondary metabolite production in *S. coelicolor* (Sulheim *et al*. 2020).

Metabolic models are not only useful as stand-alone predictive tools, but are particularly powerful when combined with the results of molecular profiling experiments, such as transcriptome or metabolome profiling (Blazier and Papin 2012; Kleessen *et al*. 2015; Graudenzi *et al*. 2018). The MORF tool (Springthorpe *et al*. 2020) can facilitate this integration by providing a single platform for interrogating multi-omics data, e.g. transcriptomics and proteomics, and then using these data as additional constraints for a linked genome-scale metabolic model.

Stoichiometric models of metabolism can be constructed from genome sequences, with very limited requirements for experimental data. This allows their application even to organisms for which data availability limits more data-demanding modelling approaches. However, ensemble modelling strategies, which allow the construction of fully parameterized kinetic models of biological systems in the absence of extensive experimental data (Tsigkinopoulou, Baker and Breitling 2017; Tsigkinopoulou *et al*. 2018), are now making it possible to model additional aspects of actinomycete biology. For example, ensemble modeling has been employed for a detailed analysis of the γ-butyrolactone network in *S. coelicolor* (Tsigkinopoulou, Takano and Breitling 2020), a system with exciting potential as an engineered regulatory circuit to control secondary metabolic pathways (Biarnes-Carrera *et al*. 2018; Biarnes-Carrera, Breitling and Takano 2018).

## REGULATORY ENGINEERING: SRNA AS AN EMERGING TOOL

Successful engineering of secondary metabolite production requires regulatory tools (Xia *et al*. 2020; Zhou, Ning and Luo 2020), which so far have not been extensively studied in actinomycetes, compared to traditional model organisms. For example, in *Streptomyces* or other antibiotic-producing actinomycetes only a few sRNA studies have been related to secondary metabolism. However, there has been much recent progress, making new regulatory tools accessible to actinomycete synthetic biologists. In *S. coelicolor*, an antisense RNA, Cnc2198.1, was found in the 5’ region of the glutamine synthetase I gene *glnA*. The controlled overexpression of this antisense RNA resulted in a decrease in growth, protein synthesis and undecylprodigiosin production (D'Alia *et al*. 2010). Another sRNA, Scr4677, is encoded in the intergenic antisense strand between two co-transcribed regulatory genes (SCO4676 and SCO4677). Whilst disruption of each regulatory gene affected actinorhodin production, modulating Scr4677 levels showed no reproducible phenotypic differences (Hindra *et al*. 2014). Mikulík *et al*. (2014) found that deletion of *ssrS* gene in *S. coelicolor*, encoding the conserved 6S RNA which binds the β and β’ subunits of RNA polymerase, reduced both the growth rate and actinorhodin production, which was accompanied by a lower expression of the pathway-specific *actII*-ORF4 transcriptional regulator (Mikulík *et al*. 2014). Using RNA-seq, Liu *et al*. (2013) identified four sRNAs that were overexpressed in an industrial erythromycin-producing strain of *Saccharopolyspora erythraea* compared to the wild type strain (Liu *et al*. 2013). These genes are encoded on the complementary DNA strands of annotated ORFs, indicating a regulatory effect on iron transport (*sernc293*), terpene metabolism (*sernc350*), geosmin synthesis (*sernc351*) and polyketide biosynthesis (*sernc389*).

These fundamental advances are very promising for future applications, but few studies of sRNA-based regulatory engineering have been published in bacteria producing secondary metabolites of industrial interest (Chaudhary, Na and Lee 2015). In *Streptomyces*, the paired termini antisense (PTas)RNA strategy has been successfully applied to downregulate an actinorhodin biosynthetic gene in *S. coelicolor* (Uguru *et al*. 2013), to enhance antibiotic production through the inhibition of the transcriptional repressor DoxR in *Streptomyces peucetius* (Chaudhary *et al*. 2015a) and to elucidate the regulatory role of the cyclic AMP receptor protein (Crp) in *Streptomyces cinnamonensis* by comparing the monensin yields and transcriptomic profiles between control, *crp*-overexpressing and *crp*-silencing strains (Lin *et al*. 2020). There is a pressing need for more extensive studies to identify sRNAs and elucidate their function in *Streptomyces* and other actinomycetes with a rich secondary metabolism.

## CLONING, REFACTORING AND ASSEMBLY OF COMPLEX METABOLIC PATHWAYS

The biosynthetic capabilities of actinomycetes are encoded in large and complex BGCs. Successful strain engineering depends on our ability to manipulate these clusters efficiently, and a wide array of *in vivo* and *in vitro* methods have been established for this purpose. *In vivo* DNA assembly methods are based on the natural homologous recombination capacity in *Saccharomyces cerevisiae* or the Red/ET homologous recombination system. Transformation-associated recombination (TAR) cloning in yeast has been successfully employed to capture and heterologously express a range of natural product BGCs, including those of taromycin A, enterocin, greocycline and cosmomycin (Yamanaka *et al*. 2014; Bonet *et al*. 2015; Bilyk *et al*. 2016; Larson, Crüsemann and Moore 2017). In a study by Eyles, Vior and Truman (2018), an integrated approach of TAR and yeast-mediated engineering was used to refactor the bottromycin BGC from *Streptomyces scabies*. After capturing the *btm* cluster in a yeast/*Escherichia coli* shuttle vector using TAR, yeast-mediated homologous recombination was employed to assemble a variety of pathway combinations using PCR products, single stranded oligonucleotides and individual pathway fragments generated by restriction digestion. This approach allowed for the simultaneous introduction of promoters, gene deletions, targeted mutations and rearrangement of gene order, yielding a refactored BGC with a 120-fold increase in bottromycin-related metabolites in the heterologous host *S. coelicolor* (Eyles, Vior and Truman 2018).

Recently, a TAR-based and CRISPR/Cas9-mediated promoter engineering platform, termed multiplex *in vitro* Cas9-TAR (*mi*CASTAR), has been developed by Kim, Rocha and Maia (2018) for the activation of the silent atolypenes BGC from *Amycolatopsis tolypomycina*. Following this approach, the *ato* cluster was digested *in vitro* by Cas9, reassembled in yeast using promoter cassettes with homology arms matching the specific Cas9 digestion sites and introduced into *Streptomyces albus* (Kim, Rocha and Maia 2018). Similar to TAR in yeast, the Red/ET homologous recombination system can be employed for assembly of complex BGCs in *E. coli*. Using Red/ET recombination, a variety of semi-synthetic promoters was incorporated into the bottromycin BGC, increasing the production titre by up to 50-fold (Horbal *et al*. 2018).

Examples of successful *in vitro* assembly methods include Gibson assembly, site-specific recombination-based tandem assembly (SSRTA) and methylation-assisted tailorable ends rational (MASTER) ligation (Zhang, Zhao and Ding 2011; Shao *et al*. 2013; Linares-Otoya *et al*. 2017). Gibson assembly is often coupled to *in vitro* applications of gene editing techniques for synthetic biology driven investigations. For example, a 36-kb jadomycin producing gene cluster from *Streptomyces venezuelae* was successfully cloned into an *E. coli* vector via Cas9-assisted targeting of chromosome segments (CATCH). RNA-guided Cas9 nuclease cleaves the target chromosome at two designated sites, prior to insertion of the target genome segment into a cloning vector by Gibson assembly. However, heterologous production of jadomycin was not reported (Jiang *et al*. 2015). In a similar manner, Li *et al*. (2017) effectively combined the CRISPR/Cas9 system with Gibson assembly to incorporate the pristinamycin II (PII) BGC into *Streptomyces pristinaespiralis*; the resulting strain produced the highest titres of PII yet reported.

Direct pathway cloning (DiPaC), a combination of long-amplicon PCR and *in vitro* DNA recombination, successfully transplanted the native erythromycin BGC into a *Streptomyces* host for heterologous expression, with production confirmed via HPLC-MS (Greunke *et al*. 2018).

In addition, *in vitro* assembly methods can also be applied in the refactoring of biosynthetic pathways for the optimized production of valuable natural products in actinomycetes. Song *et al*. (2019) utilized the ExoCET (Exonuclease combined with RecET recombination) method to construct a plasmid containing a 79 kb artificial, GC-rich cluster for the heterologous production of insecticide spinosad within *S. albus* J1074.

Often, molecular engineering tools developed in other model organisms do not immediately work when applied in actinobacteria. Ye *et al*. (2020) present an example, showing how the fine-tuning of Cas9 expression is essential to obtain good performance of a CRISPR/Cas9 system in several *Streptomyces* species.

## MANIPULATION OF PRODUCT EFFLUX FOR INCREASING YIELDS

While synthetic biology approaches have been used successfully in the engineering of metabolic pathways, resulting in bacteria that can produce high titres or novel products, the final step in the biosynthesis pathway, namely the export of the product into extracellular medium, is often overlooked, despite product efflux being a highly effective step to manipulate to increase yields in whole-cell catalysts (Kell *et al*. 2015; Lv *et al*. 2016; Soares-Silva *et al*. 2020). Most natural product BGCs in actinomycetes contain transporter genes of one type or another and their functions are almost exclusively involved in efflux, either in the secretion of the newly synthesized compound itself or in subsequent self-protection of the producing cell (Martín, Casqueiro and Liras 2005). Compared to the enzymatic machinery involved in the biosynthesis of natural products, the efflux proteins are rarely studied and only few examples of characterized systems are available, which have recently been reviewed in detail (Severi and Thomas 2019).

The simplest approach to empirically improve efflux/export is to overexpress the gene for the transporter. The streptogramin family of antibiotics provide the first example of this method, with pristinamycin produced by *Streptomyces pristinaespiralis*. The overexpression of a known resistance gene, *ptr*, that encodes an efflux pump of the major facilitator superfamily (MFS) resulted in 6 to 8-fold increase in yield of the antibiotic (Jin, Jin and Jin 2010). In a second example the production of the macrocyclic polyketide avermectin was also improved by about 50% by overexpression of the BGC-encoded ATP-binding cassette (ABC) transporter genes *avtAB* (Qiu *et al*. 2011). A third example comes from the production of oxytetracycline in *Streptomyces rimosus*, where the wild-type strain was modified to overexpress its three known self-resistance/export genes, of which two encode efflux proteins, and this resulted in oxytetracycline yields increasing over 150% (Yin *et al*. 2017).

Applying a synthetic biology approach to improve this process requires the ability to access or create variants of suitable transporter genes and apply an effective and simple high-throughput screen to assess function. An early approach of this kind was taken to try and build on the success of overexpressing *ptr* for increased pristinamycin production, where a DNA shuffling method was used to create a library of hybrid *ptr* genes taken from different high-producing strains of *S. pristinaespiralis* (Jin *et al*. 2015). However, this strategy resulted in strains producing highly variable antibiotic yields with none of the hybrids surpassing the performance of the original Ptr protein, suggesting that larger libraries of random point mutants and/or Ptr homologues from different *Streptomyces* species, such as the *varS/snbR* gene from the virginiamycin biosynthetic cluster (Mast *et al*. 2011; Severi and Thomas 2019) would be more successful.

In an excellent example of the evolutionary approach, random mutagenesis has been used to evolve a transporter with an expanded substrate range for a biotechnological application (Bali, Genee and Sommer 2018). In this work, the function of a vitamin transporter unable to take up thiamine was linked to a growth-coupled biosensor that consisted of a thiamine-responsive riboswitch silencing a selectable marker. As the starting transporter cannot import thiamine, the riboswitch represses the marker and cells die on selective medium, thus providing a powerful genetic screen for gain-of-function mutants by growth on selective media. By screening a library of 60 000 variants made by error-prone PCR on this single transporter, the authors successfully found a transporter with the desired properties. Elegant screens of this nature could be used in the context of evolving antibiotic exporters with different affinities and specificities or just increased capacity for antibiotic export. Synthetic biology approaches can be used to create these candidate parts, and in this regard the *Streptomyces* genus provides an enormous reservoir of potential antibiotic exporter genes, with many genomes encoding around 1000 different transporters (around 10% of total coding capacity; Ren, Chen and Paulsen 2007; Zhou *et al*. 2016). It is highly likely that many of these function as either specific or multi-drug exporters to provide cross-resistance to natural products produced by other species in the complex soil niche, and synthetic biology methods now enable this enormous pool of untapped transporters to be sampled, characterized and exploited.

## MOLECULAR PROFILING FOR ACTINOMYCETE SYNTHETIC BIOLOGY

Synthetic biology relies on the availability of molecular diagnostic tools for the analysis of the function of an engineered biological system. In the case of actinomycete synthetic biology, which largely focuses on the production of secondary metabolites, metabolomics is often the method of choice for this purpose. It can be applied in two major directions: either for the characterization of metabolic changes across the entire system, including primary metabolism, or to identify and quantify secondary metabolites and their precursors in a more targeted approach.

For example, a recent study on the impact of the expression of the actinorhodin BGC on the metabolome of *S. coelicolor* found pervasive effects across the metabolic network, including changes in precursors of secondary metabolites, as the result of the activity of a single biosynthetic pathway (Nitta *et al*. 2021). Quantitative analysis of the metabolome of *S. nodosus* allowed the rational engineering of improved amphotericin B production strains (Zhang *et al*. 2020).

Ideally, metabolomics is combined with other molecular profiling methods, e.g. measuring transcriptome dynamics and metabolic effects simultaneously. In actinomycete synthetic biology, such multi-omics analyses have a long tradition (Zhou *et al*. 2011; Lee *et al*. 2021), ranging from delimiting dispensable regions in *Streptomyces* genomes (Bu *et al*. 2020) and understanding the dynamic physiology of growth underlying the induction of secondary metabolite production (Kim *et al*. 2020), to understanding the effect of extracellular cAMP as a regulator of secondary metabolite production (Nitta *et al*. 2020).

Engineered actinomycetes rarely are exclusive producers of the target chemical. More often, they co-produce relatively complex mixtures of secondary metabolites, creating additional analytical challenges for metabolomics profiling. Recent advances in LC-MS based approaches and tools for the fast identification of natural products, such as Global Natural Products Social molecular networking (GNPS) have revolutionized natural product research (Wang *et al*. 2016). Yet, the challenge remains of annotating the spectral information and identifying detected metabolites by linking isotopic patterns and fragmentation data to given molecular structures in secondary metabolites databases (Allard, Genta-Jouve and Wolfender 2017; Caesar *et al*. 2019). In the case of comprehensive metabolome profiles, Bayesian methods that take into account the plausible biochemical relationships among the ingredients of a sample can add further support to the annotation process—and provide a principled statistical assessment of the confidence in individual identifications (Tripathi *et al*. 2021).

Despite considerable advances, the structural elucidation of co-produced natural products remains a complex process leading in some cases to the assignment of incorrect structures. Computer Assisted Structural Elucidation (CASE) approaches, both commercial and academic, have been developed as powerful tools to help in the process through the generation of prioritized structural proposals, although these applications provide only planar (2D) structures and further improvements are still required to determine 3D structures (Burns, Mazzola and Reynolds 2019). Moreover, the DP4-AI software has emerged as a tool complementary to CASE for completely automated 3D structural elucidation starting from raw ^13^C and ^1^H NMR data (Howarth, Ermanis and Goodman 2020). Apart from these tools, databases including calculated/real NMR data and NMR structural features easily recognizable in NMR spectra have been developed to speed up the structural elucidation of new compounds and were reviewed recently (Pérez-Victoria, Martín and Reyes 2016).

Since then, other approaches based on the use of NMR in combination with structural features obtained from fast NMR experiments (Zani and Carroll 2017) or 2D heteronuclear single quantum coherence (HSQC) NMR spectra in combination with MS or ^1^H NMR data have been developed. Perhaps the most remarkable effort in this sense has been the Small Molecule Accurate Recognition Technology (SMART) initiative (Reher *et al*. 2020). Using the HSQC spectrum of a sample as a query, this Artificial Intelligence-based tool allows the generation of structural hypotheses by comparative analysis with a library of > 100,000 real or computed spectra of natural products. The system also includes external links to other platforms including mass spectrometry, BGC and structural information in cases where known natural products are identified: GNPS, MiBIG (Kautsar *et al*. 2020) and the Natural Products Atlas (Van Santen *et al*. 2019). Lately, ^13^C NMR-based tools have also been developed to dereplicate compounds in mixtures (MixONat; Bruguière *et al*. 2020) or the prediction of natural product classes (Martínez-Treviño *et al*. 2020).

## APPLICATION CASE 1: CHALLENGES OF TETRACYCLINE PRODUCTION

The native tetracycline (TC) producing strains *Streptomyces aureofaciens* and *S. rimosus* are powerful industrial producers of chlortetracycline (CTC) and oxytetracycline (OTC), respectively, aromatic polyketide metabolites of medical and agricultural importance (Chopra and Roberts 2001). Industrial strains for the production of these valuable compounds have been developed over many decades by intensive mutagenesis and strain selection approaches (Goodman 1985). The main challenge today is to further improve the yield of target TCs, particularly by increasing the rate of conversion of the carbon source, mostly from media containing starch, while reducing the presence of undesired impurities. OTC-producing *S. rimosus* strains are generally easily transformable, fast-growing strains compared to many other streptomycetes and numerous industrially useful molecular biology tools and microbiology methods are available, including the latest genome editing technologies, which make these strains attractive chassis for a synthetic biology approach (Jia *et al*. 2017; Carrillo Rincón *et al*. 2018).

Early successes were realized when a chlortetracycline BGC was heterologously expressed in the industrial strain *S. rimosus* 461, and additional engineering to achieve overexpression of a cluster-situated activator led to 38-fold increase in titre of CTC compared to the original producer (Wang *et al*. 2019). There is at present a critical need for novel antibiotics, to address the rapid spread of antibacterial resistance, and TC intermediates and shunt products may represent a very attractive source of drug leads (Martens and Demain 2017; Petković, Lukežič and Šušković 2017). Because of the rapid development of synthetic biology approaches, it is now possible to generate novel TC scaffolds through rational approaches (Wang *et al*. 2012; Cummings, Breitling and Takano 2014; Lukežič *et al*. 2019). An important successful example is provided by the work of Wang *et al*. (2012) who used three structurally diverse natural tetracycline BGCs encoding antibiotic OTC, the antitumor SF2575 and dactylocycline as the starting point for engineering. A number of new tetracycline compounds were obtained from the engineered host-pathway pairs (Wang *et al*. 2012). Another example is the introduction of a novel starter unit into the backbone of the atypical tetracycline chelocardin produced by *Amycolatopsis sulphurea*, which results in a potent antibacterial compound currently in preclinical evaluation (Lešnik *et al*. 2015).

However, it is becoming clear that polyketide synthase (PKS) enzymatic complexes catalysing the formation of TCs are not very robust, i.e. even a small modification of the targeted enzyme(s) may inhibit the catalytic activity of these complex enzymes, consequently disabling the functionality of the entire PKS complex. Most often, this results in a significant drop in the titres of target intermediate(s) or even in a derailment of the whole Type II PKS complex. Moreover, we have not yet completely elucidated the preferred order of the enzymatic reactions, particularly those related to the late stages in TC biosynthesis (post-PKS biosynthesis), when functional groups are added to the basic polyketide backbone (Wang *et al*. 2013). To fully capitalize on the potential of synthetic biology for improved and diversified tetracycline production, there is a need for more in-depth understanding of the catalytic mechanisms of the enzyme complexes involved.

## APPLICATION CASE 2: PRODUCING RIPPS

Ribosomally synthesized and posttranslationally modified peptides (RIPPs) are one of the major groups of bioactive compounds found in actinomycetes. Because of its well-studied genetic background and a plethora of available molecular tools for strain engineering, *S. coelicolor* is one of the most frequently used hosts for heterologous expression of RiPP BGCs. Thiostreptamide S4, which belongs to a class of structurally novel RiPPs characterized by a contiguous sequence of thioamide groups, was originally isolated from *Streptomyces* sp. NRRL S-4 and heterologously produced in *S. coelicolor* M1146 at levels comparable to the native producer (Frattaruolo *et al*. 2017). Similarly, thiovarsolins, a second class of thioamidated RiPPs, were discovered in the genetically intractable *S. varsoviensis* and heterologously produced in *S. coelicolor* M1146 (Santos-Aberturas *et al*. 2019). In case of the leepeptin BGC from *S. leeuwenhoekii* C34T, constitutive expression of the BGC in the heterologous host *S. coelicolor* was necessary for sufficient quantities of leepeptin for enabling structural elucidation (Gomez-Escribano *et al*. 2019).

Aside from *S. coelicolor*, a variety of RiPP-encoding BGCs have been successfully expressed in other heterologous *Streptomyces* hosts in recent years. Using heterologous expression, it was possible to increase production of MS-271 18-fold and confirm that epimerization of the C-terminal L-tryptophan into its D-form involves a novel, uncharacterized epimerase (Feng *et al*. 2018). *S. lividans* TK24 has been also employed to produce a library of natural and designed derivatives of thioviridamide/neothioviridamide, an anti-cancer agent of RiPPs origin with polythioamide backbone. A total of eight of the designed molecules exhibit cytotoxicity twice that of the wild-type natural product (Kudo *et al*. 2019). Derivatization of the RiPP cinnamycin was achieved by incorporation of three noncanonical amino acid residues into two different positions of the core peptide. The methodology was developed using *S. albus* as a heterologous host via co-expression of the *cin* BGC and *pyl*RS/tRNA^Pyl^ pair. In *S. albus*, the titre of deoxycinnamycin produced was 5-fold greater relative to the natural mixture of cinnamycins produced by the same heterologous host (Lopatniuk, Myronovskyi and Luzhetskyy 2017).

As the metabolic engineering of actinomycetes is frequently challenging, heterologous expression in *E. coli* is an important tool when probing the mechanisms of RiPP biosynthesis pathways derived from actinomycetes (Hwang *et al*. 2014). For example, a recent study investigating the installation of azole rings within the sulfomycin biosynthesis pathway of *S. viridochromogenes* employed *E. coli* as part of a heterologous expression strategy to examine the roles of a number of key residues within the core peptide of precursor peptide, SulA (Du *et al*. 2020). In addition, the characterization of a novel venezuelin-like lanthipeptide was enabled by the establishment of an *E. coli*-based heterologous production system. Consequently, the study provided greater insight into the mechanism of class IV lanthipeptide biosynthesis (Hegemann and Van Der Donk 2018). The discovery of a unique two-component lantibiotic, roseocin, was in part facilitated by the partial *in vitro* reconstitution of the cluster within *E. coli*. The resulting posttranslationally modified lanthipeptides (RosA1 and RosA2) obtained from heterologous expression within *E. coli* were subsequently converted into bioactive roseocin via *in vitro* cleavage using commercial proteases (Singh, Chaudhary and Sareen 2020).

## CONCLUSION

The recent great technological advances in the area of synthetic biology of actinomycetes present a great promise for a revolutionary transformation of the biotechnological production of powerful new drugs. They can facilitate the biochemical characterization of new compounds, help enhance their production to economically viable levels, support the chemical diversification of initial chemical scaffolds, and accelerate the transfer of new compounds to industrial production. The examples discussed illustrate the range of the techniques and concepts that contribute to this technological revolution, but they also highlight the numerous areas that still require further research before the full potential of synthetic biology can be realized. Actinomycetes, with their immense chemical diversity and their unique challenges and opportunities for biosynthetic engineering, will remain at the forefront of these developments.
